# Supplementary material for: Humidity-tolerant porous polymer coating for passive daytime radiative cooling
Source: Nat Commun. 2024 May 25;15:4457. doi: 10.1038/s41467-024-48621-6 (PMC11127965; doi:10.1038/s41467-024-48621-6)
Supplement: Supplementary file 1 — Supplementary Information [file 41467_2024_48621_MOESM1_ESM.pdf]

Supplementary Information for

Humidity-tolerant porous polymer coating for passive daytime radiative cooling

Dongpyo Hong[1†], Yong Joon Lee[2†], Ok Sung Jeon[1], In-Sung Lee[1], Se Hun Lee[1], Jae Yeon Won[3], Young Pyo Jeon[1], Yunju La[1], Seonmyeong Kim[4,5], Gun-Sik Park[4], Young Joon Yoo[1] & Sang Yoon Park[1,3*]*

[1]Advanced Institute of Convergence Technology, Seoul National University, Gyeonggi-do 16229, Republic of Korea

[2]PURITECH co., Ltd., Gyeonggi-do 17745, Republic of Korea

[3]School of Electronic Engineering, Kyounggi University, Gyeonggi-do 16227, Republic of Korea

[4]Center for THz-Driven Biomedical Systems, Department of Physics and Astronomy, Institute of Applied Physics, College of Natural Sciences, Seoul National University, Seoul 08826, Republic of Korea

[5]Mechatronics Research, Samsung Electronics Co., Ltd., Hwaseong-si, Gyeonggi-do 18448, Republic of Korea

[†] These authors equally contributed to this work.

*Corresponding author e-mail addresses: youngjoonyoo@snu.ac.kr, yoonpark@kgu.ac.kr

This supplementary information contains the following sections:

Supplementary Notes

Supplementary Fig 1-8

Supplementary References

Supplementary note

Finite-Difference Time-Domain Simulations for isolated pore.

To investigate the scattering characteristics of individual pores depending on morphology, Finite-Difference Time-Domain (FDTD) simulations were carried out using Ansys Lumerical 2022. The size of unit cell was set as 3 times of diameter of the pore, and mesh dimension was set as 10×10×10 nm. To calculate scattering efficiency and scattering cross-section, total-field scattered-field source and DFT monitors on every boundary plane were used, and the wavelength was set as 0.28 ~ 2.5 μm to simulate scattering of solar lights. In this scattering system, the absorption by base medium was ignored, and the refractive index of it was considered as 1.49. The aspect ratio of each spheroidal pore was changed 1 to 4 while the volume of each model was maintained same, because the simulation focused on the dependency of pore morphology.

Diffusion model

The use of a diffusion model is a well-established approach for describing electromagnetic propagation through disordered media.[1] In this model, universal transport through the medium is described by superposition of a large number of single scattering events. When the absorption is weak ($\alpha t \ll 1$), the asymptotic form for wave transmittance through the non-absorptive diffusive slab can be represented as follows:[2]

$$T(t) = 5\alpha D/v\sinh(\alpha t) \approx 5D/vt \quad (1)$$

where $T(t)$ is the transmittance of a slab with thickness t, D is the diffusion coefficient, and v is the wave velocity, α is absorption coefficient. The diffusion coefficient can be represented in terms of the transport mean free path l in which the direction of propagation is randomized:

$$D = vl/3 \tag{2}$$

$$l = \rho^{-1}\sigma^{-1}(1 - <\cos\theta>)^{-1} \tag{3}$$

where ρ is the number density, σ is the scattering cross-section, and $<\cos\theta>$ is the average cosine of the scattered angle, weighted by scattering function. Hence, the transmittance of the slab can also be written as follows:

$$T(t) = (5/3)\,\rho_s^{-1}\,\sigma^{-1}(1 - <\cos\theta>)^{-1} \tag{4}$$

where $\rho_s(=\rho/t)$ is the areal number density of scattereres. Since the scattering cross-section and $<\cos\theta>$ can be extracted through a numerical simulation for a single pore, this formalism allows for a prediction of the transmittance trend according to changes in the pore structure. Assuming negligible absorption as in actual PVDF-HFP, the calculated transmittance was compared with the experimental result as 1-Reflectance.

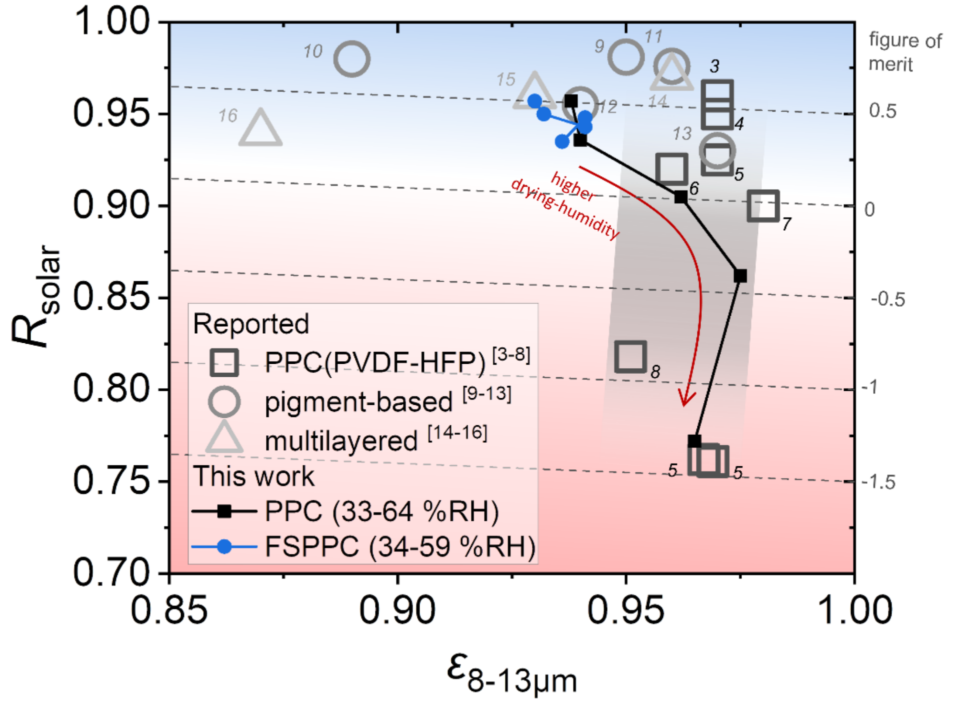

Supplementary Fig. 1. Optical performance of previously reported PDRC materials including porous polymer coating (PPC)[3-8], pigment-based radiative cooler[9-13], radiative cooler with multilayered structure[14-16]. The optical properties of PPC and FSPPC with different drying-humidity measured in this study are displayed as solid lines with filled symbol. The dash line is the contour line of the figure of merit described in the text. Region with negative and positive figure of merit are colored by red and blue respectively. Large deviation of the PPC is emphasized by gray shadow.

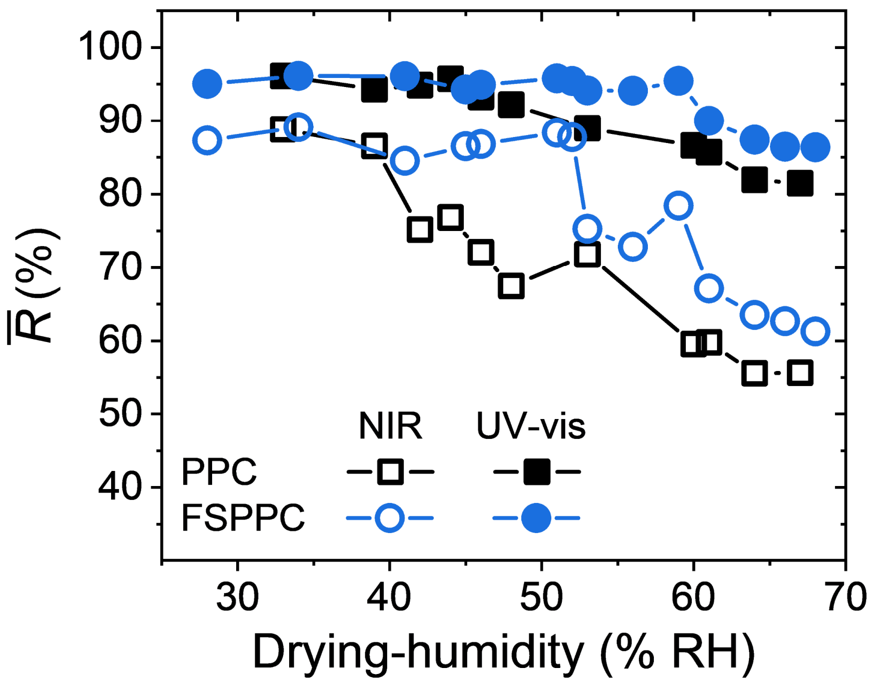

Supplementary Fig. 2. Averaged reflectance of PPC and FSPPC in the spectral range of UV-vis (0.28-0.75 μm) and NIR (0.75-2.5 μm).

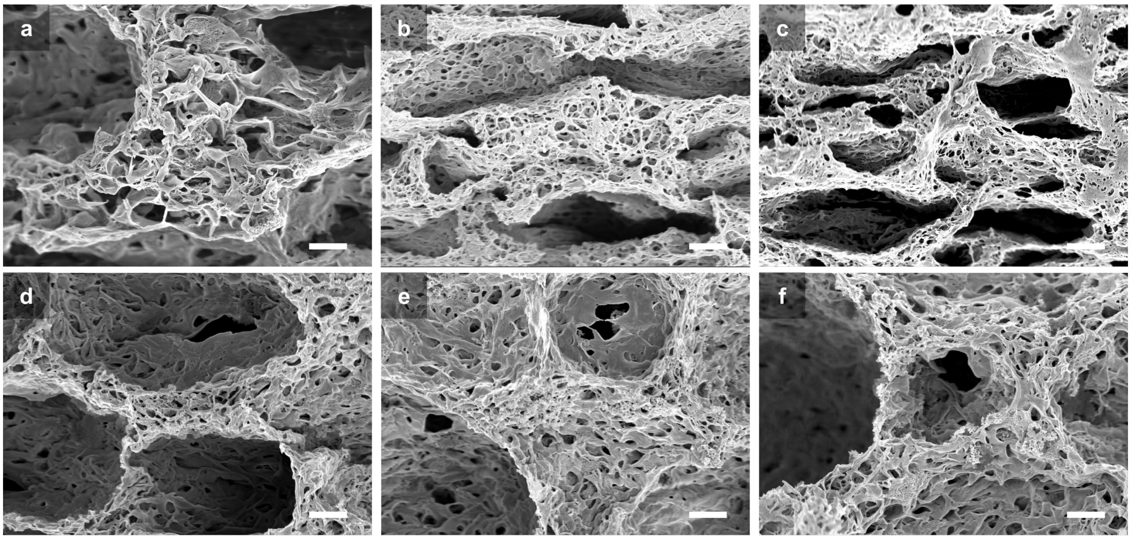

Supplementary Fig. 3. High magnification SEM cross-section images for nanoporous structure of PPC((a) 33, (b) 46, (c) 64% RH) and FSPPC ((d) 34, (e) 46, (f) 59% RH) dried at different humidity. All the scale bars are 1μm.

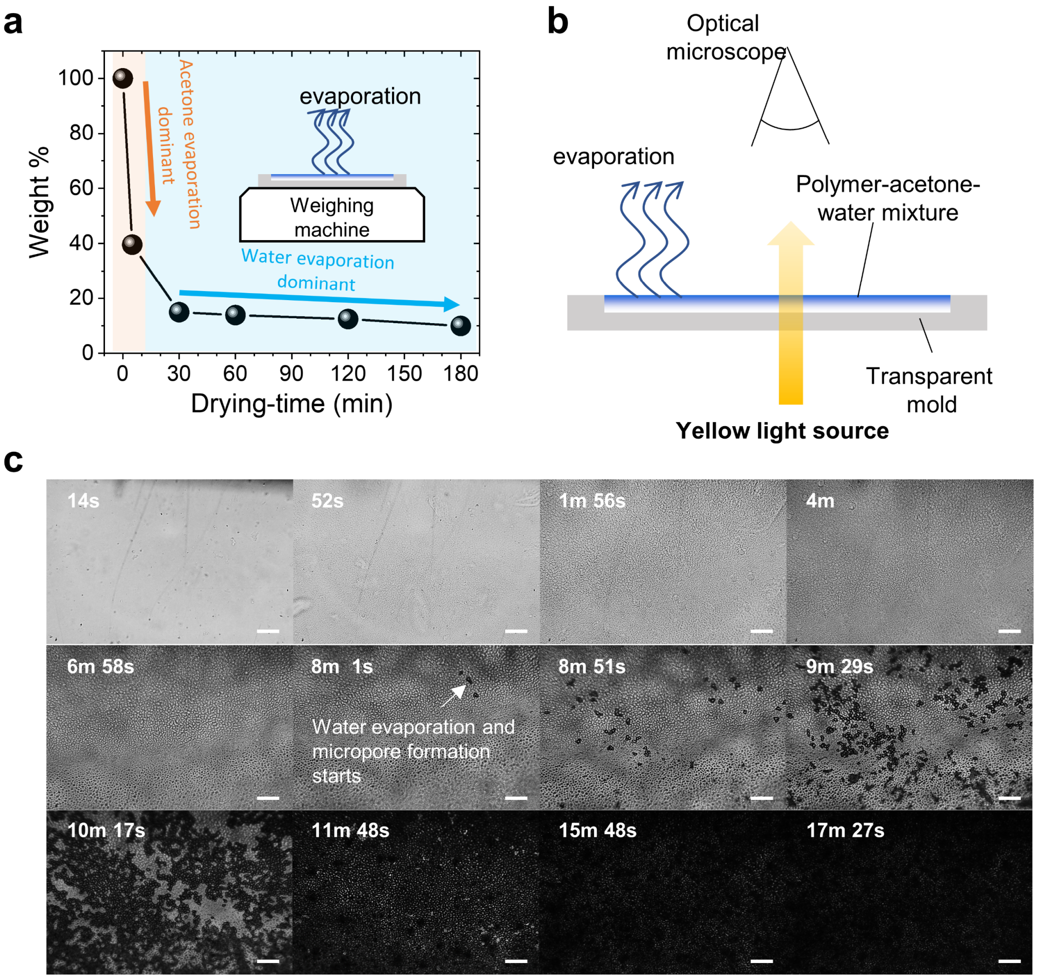

Supplementary Fig. 4. Gravimetric and optical in-situ analysis of PPC during drying-process. (a) Normalized weight change of mixture according to drying time. The phase where acetone is mainly evaporated is colored orange, and the section where water is mainly evaporated is colored light blue. (b) Schematic of experimental setup for in-situ drying observation of PPC. (c) Optical microscope images at different drying times.

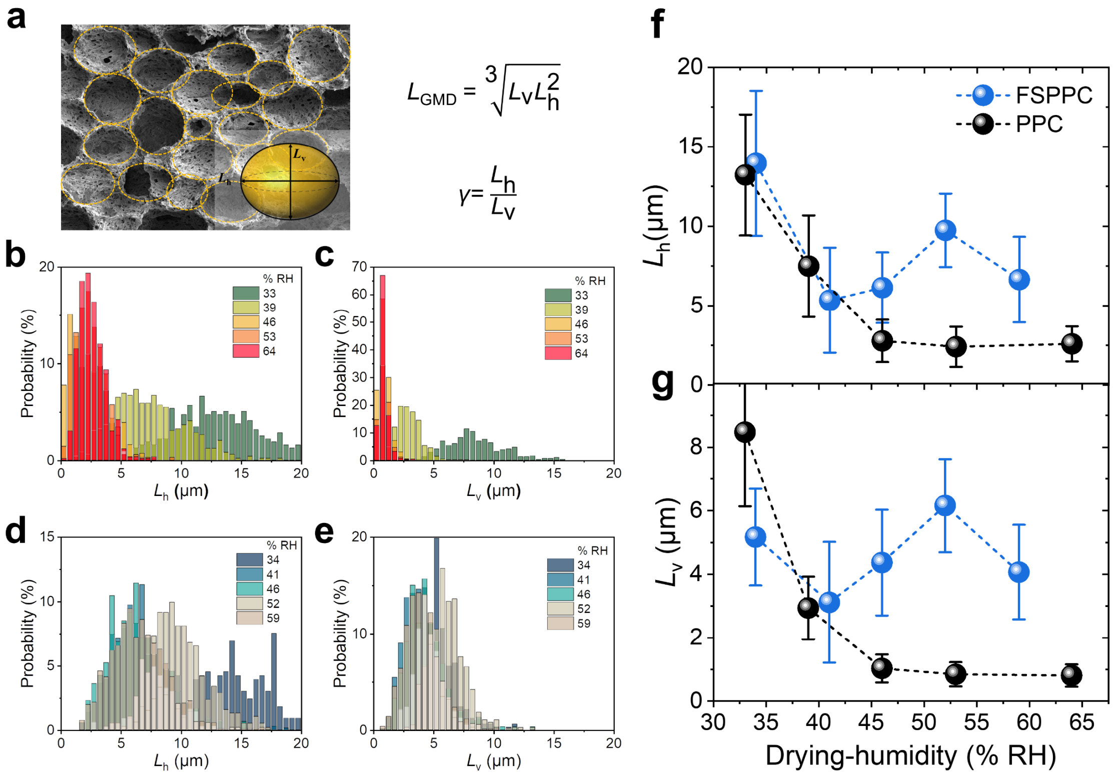

Supplementary Fig. 5. Statistical analysis of micropore size distribution in PPC and FSPPC dried at different humidity. a) Spheroidal modelling of internal pores and definition of L_h and L_v. (b-e) Statistical distribution of L_h and L_v for PPC (b-c) and FSPPC (d-e). (f-g) Averaged L_h and L_v of PPC and FSPPC depending on the drying-humidity. Error bar means standard deviation of the size distribution. Analysis was conducted on more than 300 pores for each sample.

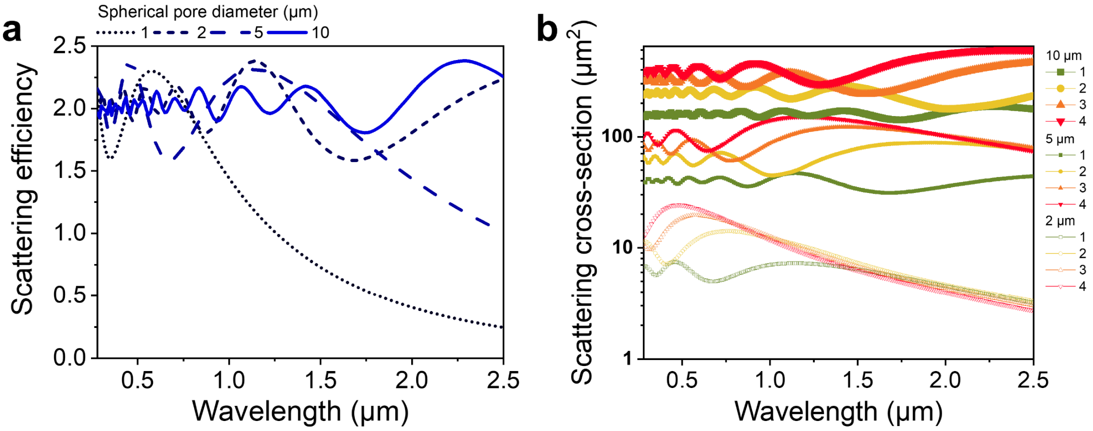

Supplementary Fig. 6. Scattering characteristics of individual pore depending on its size and shape. (a) Scattering efficiency of spherical pore in the solar spectral range calculated by FDTD simulation. (b) Scattering cross-section of a spheroidal pore with various L_{GMD} and γ.

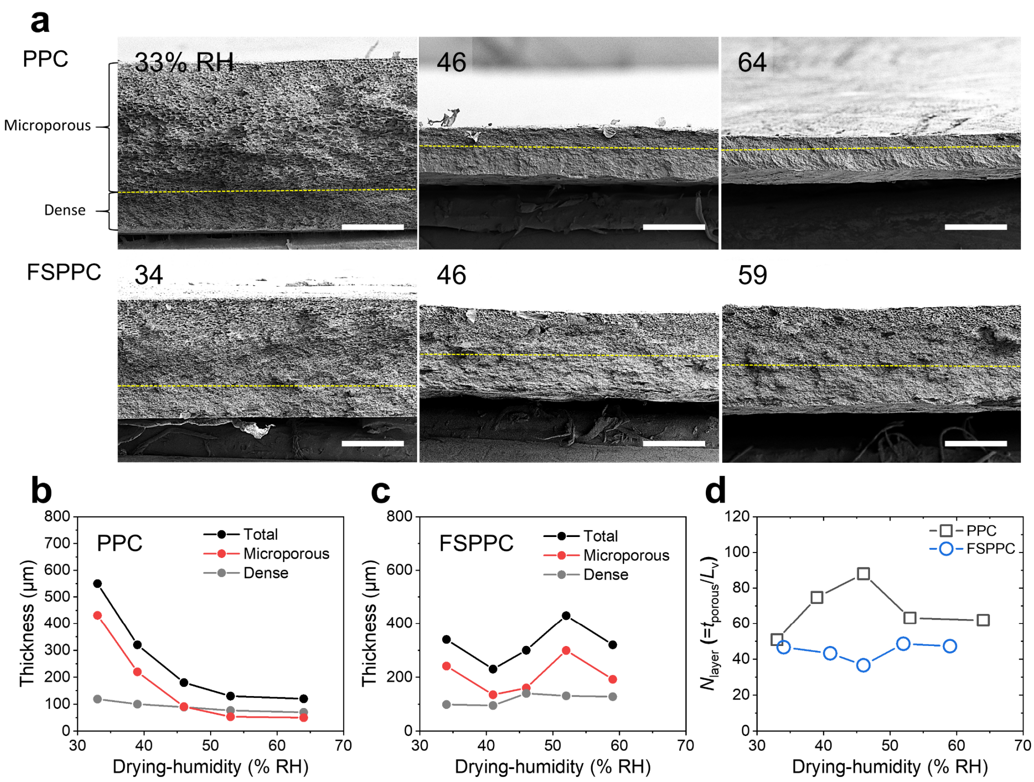

Supplementary Fig. 7. Asymmetric morphology of PPC and FSPPC. (a) low-magnification cross-section SEM image for PPC and FSPPC dried at different humidity. All scalebars are 200 μm. The film shows asymmetric nature where boundary dividing microporous region and dense region is emphasized by yellow dash line. Thickness of microporous upper region, dense lower region and total film of PPC (b) and FSPPC (c) dried at different humidity. (d) Number of pore layer calculated by dividing thickness of microporous region by average vertical diameter of micropore in Supplementary Fig. 5.

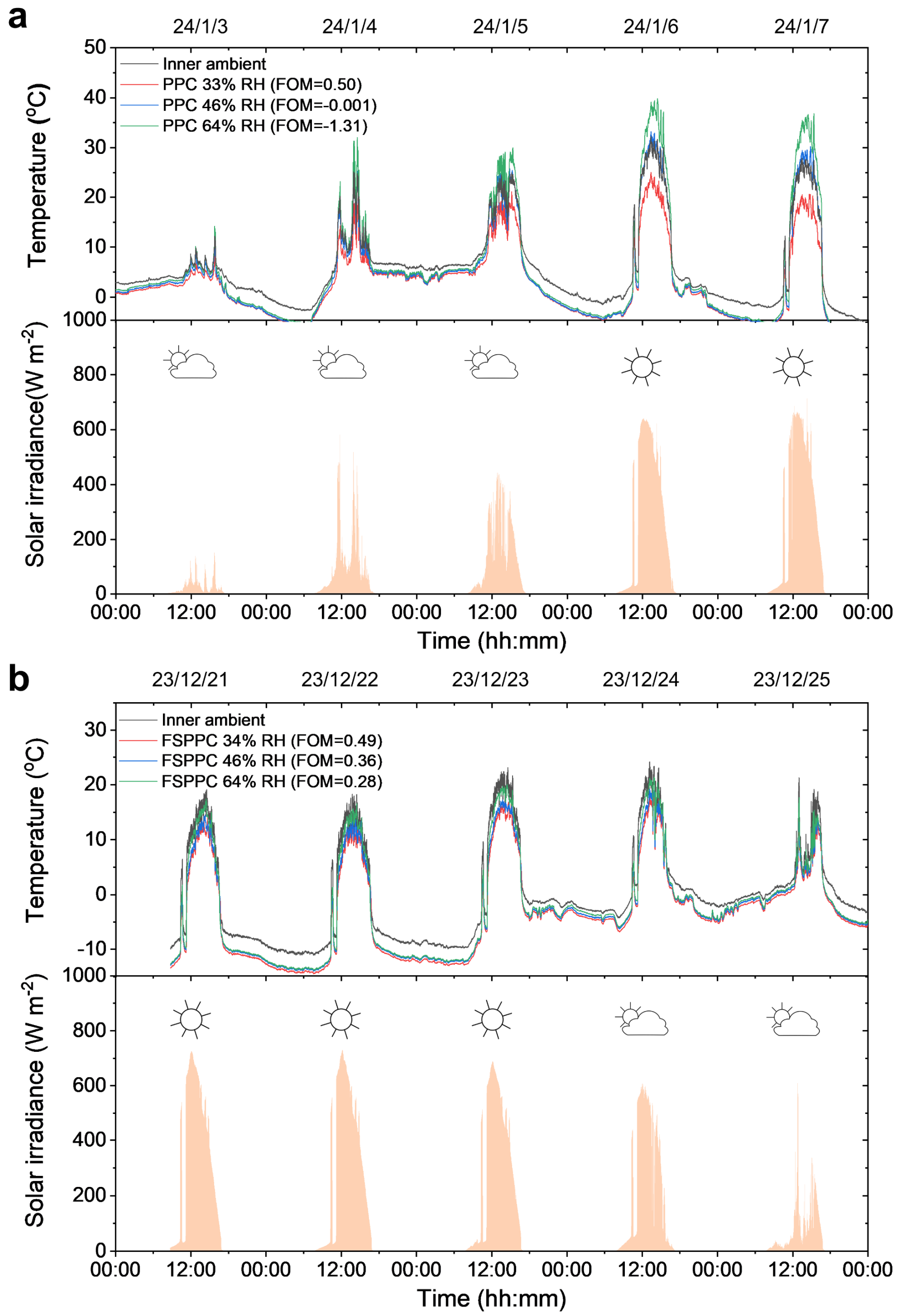

Supplementary Fig. 8. Simultaneous field test of PPC (a) and FSPPC (b) with various drying-humidity for consecutive five days.

Supplementary References

(1) Ishimaru, A. *Wave propagation and scattering in random media*; Academic press New York, 1978.
(2) Genack, A. Z. Optical transmission in disordered media. *Phys. Rev. Lett.* **1987**, *58* (20), 2043-2046. DOI: 10.1103/PhysRevLett.58.2043 From NLM Publisher.
(3) Mandal, J.; Fu, Y.; Overvig, A. C.; Jia, M.; Sun, K.; Shi, N. N.; Zhou, H.; Xiao, X.; Yu, N.; Yang, Y. Hierarchically porous polymer coatings for highly efficient passive daytime radiative cooling. *Science* **2018**, *362* (6412), 315-319.
(4) Xue, C.-H.; Wei, R.-X.; Guo, X.-J.; Liu, B.-Y.; Du, M.-M.; Huang, M.-C.; Li, H.-G.; Jia, S.-T. Fabrication of superhydrophobic P (VDF-HFP)/SiO2 composite film for stable radiative cooling. *Compos. Sci. Technol.* **2022**, *220*, 109279.
(5) Zhang, H.; Huang, J.; Fan, D.; Tie, P. Inverse design, fabrication, and tolerance to extreme environments of radiative cooling coating. *Optical Materials Express* **2021**, *11* (11), 3706-3716.
(6) Zhu, J.; An, Z.; Zhang, A.; Du, Y.; Zhou, X.; Geng, Y.; Chen, G. Anisotropic porous designed polymer coatings for high-performance passive all-day radiative cooling. *Iscience* **2022**, *25* (4), 104126.
(7) Zhang, H.; Fan, D. Improving heat dissipation and temperature uniformity in radiative cooling coating. *Energy Technology* **2020**, *8* (5), 1901362.
(8) Ma, H.; Wang, L.; Dou, S.; Zhao, H.; Huang, M.; Xu, Z.; Zhang, X.; Xu, X.; Zhang, A.; Yue, H. Flexible daytime radiative cooling enhanced by enabling three-phase composites with scattering interfaces between silica microspheres and hierarchical porous coatings. *ACS Appl. Mater. Interfaces* **2021**, *13* (16), 19282-19290.
(9) Li, X.; Peoples, J.; Yao, P.; Ruan, X. Ultrawhite BaSO4 paints and films for remarkable daytime subambient radiative cooling. *ACS Appl. Mater. Interfaces* **2021**, *13* (18), 21733-21739.
(10) Li, P.; Wang, A.; Fan, J.; Kang, Q.; Jiang, P.; Bao, H.; Huang, X. Thermo-optically designed scalable photonic films with high thermal conductivity for subambient and above-ambient radiative cooling. *Adv. Funct. Mater.* **2022**, *32* (5), 2109542.
(11) Lv, J.; Chen, Z.; Li, X. Calcium phosphate paints for full-daytime subambient radiative cooling. *ACS Applied Energy Materials* **2022**, *5* (4), 4117-4124.
(12) Li, X.; Peoples, J.; Huang, Z.; Zhao, Z.; Qiu, J.; Ruan, X. Full daytime sub-ambient radiative cooling in commercial-like paints with high figure of merit. *Cell Rep. Phys. Sci.* **2020**, *1* (10), 100221.
(13) Song, J.; Zhang, W.; Sun, Z.; Pan, M.; Tian, F.; Li, X.; Ye, M.; Deng, X. Durable radiative cooling against environmental aging. *Nat. Commun.* **2022**, *13* (1), 4805.
(14) Gentle, A. R.; Smith, G. B. A subambient open roof surface under the Mid-Summer sun. *Adv. Sci.* **2015**, *2* (9).
(15) Haechler, I.; Park, H.; Schnoering, G.; Gulich, T.; Rohner, M.; Tripathy, A.; Milionis, A.; Schutzius, T. M.; Poulikakos, D. Exploiting radiative cooling for uninterrupted 24-hour water harvesting from the atmosphere. *Sci. Adv.* **2021**, *7* (26), eabf3978.
(16) Chae, D.; Kim, M.; Jung, P.-H.; Son, S.; Seo, J.; Liu, Y.; Lee, B. J.; Lee, H. Spectrally selective inorganic-based multilayer emitter for daytime radiative cooling. *ACS Appl. Mater. Interfaces* **2020**, *12* (7), 8073-8081.
